# Loneliness, Social Support, Social Trust, and Subjective Wellness in Low-Income Children: A Longitudinal Approach

**DOI:** 10.3390/children10091433

**Published:** 2023-08-23

**Authors:** Hanife Akgül, Ahmet Zeki Güven, Sibel Güven, Müyesser Ceylan

**Affiliations:** 1 Faculty of Education, Çanakkale Onsekiz Mart University, Çanakkale 17100, Türkiye; hanifeakgul@comu.edu.tr (H.A.); s_guven@comu.edu.tr (S.G.); 2 Faculty of Education, Department of Turkish Language Education, Akdeniz University, Antalya 07070, Türkiye; 3 Faculty of Education, Anadolu University, Eskisehir 26470, Türkiye; mceylan@anadolu.edu.tr

**Keywords:** loneliness, children, social support, social trust, wellness

## Abstract

The progress and development of society in every sense is possible by raising healthy individuals. To do so, it is necessary to ensure the physical and mental development of children in a healthy way. There are many variables that affect the physical and mental development of children. These variables are affected by individual factors, social structure, social interactions and cultural values. In addition, when these factors interact with each other, their effect on behavior and well-being may increase. Undoubtedly, one of the primary factors negatively affecting a child’s physical and psychological development is the adverse economic conditions and hardships experienced by his/her family and consequently, by the child. Increasing poverty hinders children’s access to resources, and thus negatively affects their mental health as well as their physical development. Furthermore, positive economic conditions pave the way for an improved environment, better nutrition, higher-quality education, elevated social status, more friends, reduced feelings of loneliness, and increased social support and trust and all of these positively contribute to psychological well-being. Therefore, based on the conviction that early interventions can be protective and screening is needed to determine the proper intervention, this study aims to investigate the relationship between psychological well-being, loneliness, social support and social trust, all of which affect the psychological health of children living in economically disadvantaged families. To this end, answers to the following questions were sought. Is there a significant relationship between the loneliness, social support, social trust and psychological well-being of the children from low-income families? Do the feelings of loneliness, social support and social trust of the children from low-income families significantly predict their psychological well-being?

## 1. Introduction

A healthy society is possible with healthy individuals. The World Health Organization defines the concept of “health” as a state of complete physical, mental and social well-being, not just the absence of disease or disability. For this reason, it is emphasized that the concept of psychological well-being as the basis of health is indispensable and that psychological well-being, including physical well-being, is also valuable [1].

Children are the guarantee of the continuation of society. It is important that children have a healthy childhood for the continuity of a healthy society. Childhood is a critical period that affects all periods of life and shapes the mental and spiritual structure of the individual. In this context, the roles of education policies and family institutions are important in ensuring both the mental and psychological well-being of children. Examining and studying the psychological health of children is important in terms of providing opportunities for prevention and improvement, such as early diagnosis/treatment of mental health problems experienced in adulthood. Examining and studying the psychological health of children will also serve as a prevention work by ensuring that childhood is lived happily and healthily [2,3,4,5,6,7,8,9,10]. 

Loneliness, as a concept affecting children’s well-being, is a feeling that every person can feel. Loneliness is both objective and subjective. Although loneliness literally means “the state of having no one”, in fact, an individual may feel lonely in crowds. On the other hand, an individual who is alone may not feel lonely. Some people enjoy solitude [11]. It is important to examine the emotional state of the individual here. What meanings he/she attributes to this feeling and what these meanings make him/her feel will best explain the concept of loneliness. The concept of loneliness is, therefore, divided into the meaning of the word and the meaning felt. The feeling of loneliness can be said to be a psychologically unpleasant situation. When the literature is examined, loneliness is generally defined as a distressing, painful and mental health-affecting experience with potentially severe consequences [12,13,14,15]. According to Sullivan [16], loneliness is the most painful of all human experiences, arising when a person’s need for intimacy is not met. Peplau and Perlman [13], define loneliness as “an unpleasant psychological state” that occurs in one way or another, quantitatively or qualitatively, in one’s social relationships. Although there are different definitions reflecting the theoretical foundations, there are common points in the definitions. When the definitions of loneliness are examined, the common points identified are as follows: Loneliness is an experience that affects people, it is an unwanted and painful feeling, it is an unpleasant experience that affects psychological well-being, it is closely related to social relations, it is a subjective experience [12,15,17,18].

Research has shown that loneliness, which is associated with various psychological and physical disorders [19,20,21], is linked to alcohol dependence, insatiability, suicidal tendencies, psychosomatic illnesses, life satisfaction, self-esteem, unhappiness, anxiety, shame, alienation, isolation, depression and delinquency [22]. From a young age, the mental and spiritual structure of the individual begins to take shape. For a child to be able to live in harmony with the environment, regulate himself/herself and his/her environment, exhibit healthy development and thus lead a happier life, it is essential that not only his/her physical health but also his/her mental health should be good. Psychological well-being is an essential concept for a healthy personality. 

Many studies consider trust as an indicator of a healthy personality and attribute a key role to it in interpersonal and social relationships [23,24,25]. The feeling of loneliness has been increasing in recent years and is one of the prominent emotions that negatively affect children’s mental health [26]. As societies change and technology advances, individuals and children are experiencing increasing levels of isolation, leading loneliness to become one of the most intense emotions that people have been experiencing lately [22]. In fact, loneliness, which is a natural feeling for every person is an unpleasant mood that hurts the individual whether he/she is a child, an adolescent, an adult or elderly and negatively affects psychological well-being. For this reason, there is a negative relationship between the feeling of loneliness and the well-being of the individual. The lonelier the individual becomes, the more he/she will be negatively affected physically and mentally. Studies have shown that more than half of the people with severe mental illness are lonely [27]. In the study conducted by Rokach [28], on loneliness, it was stated that loneliness harms individuals’ lives by depleting their energy and directing them towards negative activities. Loneliness was found to damage a person’s feelings of love and closeness, replacing them with jealousy and possessiveness, thereby causing harm to social and close relationships. As a consequence, individuals become deprived of social support networks, leading to isolation. One of the factors affecting the well-being of the individual is social support. 

The strengthening of the bonds of love and affection with one’s surroundings during moments of crisis is referred to as social support [29]. In this context, fundamentally, an individual is not a solitary entity but a “social” being, whose life gains meaning through social interactions and is supported by various factors within these interactions [30]. According to Yıldırım [31], the first close circle of the individual consists primarily of his/her family, friends and special people he/she knows. While the psychological and social assistance obtained from this close circle is defined as social support, the resources provided by this close circle are referred to as the main resources of social support. Children’s well-being is positively or negatively affected by their interaction with their environment [32]. It has also been found that there is a close link between childhood well-being and positive family relationships [10,33]. In addition, it is thought that the family has a central role in assessing children’s well-being and that each parent is an expert on their child’s strengths, abilities, tendencies, and competencies, which makes it a more reliable data source. In the literature, it is explained that social support has significant effects on psychological and physical well-being, and in its absence, various problems can arise. Social support is considered to play a crucial role in the resolution of these problems [31]. It has been reported that individuals who receive social support from their own environmental resources tend to have increased self-confidence and display positive behaviors within the community while experiencing a decrease in negative psychological symptoms [34]. Particularly, the individual will overcome the negative emotions he/she experiences in the face of loneliness through the social support he/she receives from the people in his/her close circle, leading to a more qualitatively improved state of well-being. One of the factors affecting the well-being of the individual is social trust. 

Social trust which is one of the most fundamental determinants in interpersonal relationships plays a critical role in the establishment and continuity of human interactions, as well as in the process of social support. Social trust is an emotion that encompasses both optimism and fear; this fear operates in conjunction with the feelings of making mistakes and embarrassment. Since social trust is an abstract concept, it brings with it the obligation to act under the influence of feelings in relationships. In the process of developing mutual trust, individuals are influenced by attitudes, behaviors, expectations and the form of social relationships [35]. According to the World Values Survey conducted between 2010 and 2014, the level of trust among adult individuals towards each other is reported to be 12%, while there are no available data for children [35]. According to the Values Survey, the common feature of societies with the highest level of trust is that they are wealthy and welfare societies. There are two main claims about the sources of trust. According to the first claim, trust is an individual state developing through various influences but encoded in the character. The other claim is that trust is a concept that is shaped based on the individual’s perception of the external environment and other people, and it is not about the individual himself/herself but rather about the environment outside the individual. Rotter’s point of view is an approach grounded in the science of psychology. Rotter [36], stated that all areas of our daily life and social life are built on social trust. Studies have shown that psychological well-being is very important for a healthy personality, and trust is accepted as one of the important indicators of a healthy personality. For this reason, trust plays a key role in interpersonal and social relations [23,24,25].

The progress and development of society in every sense is possible by raising healthy individuals. To do so, it is necessary to ensure the physical and mental development of children in a healthy way. There are many variables that affect the physical and mental development of children. These variables are affected by individual factors, social structure, social interactions and cultural values [37]. Therefore, what happens in daily life, at home, at school and on the street affects the mental health of the child [38]. In addition, when these factors interact with each other, their effect on behavior and well-being may increase. For example, factors such as unemployment, low income and limited education of a child’s parents, along with a distressing school environment, can increase negative emotions such as anger, anxiety and loneliness in the child, leading to deterioration in the child’s psychological well-being [39]. Undoubtedly, one of the primary factors negatively affecting a child’s physical and psychological development is the adverse economic conditions and hardships experienced by his/her family and consequently, by the child [40]. Increasing poverty hinders children’s access to resources, and thus negatively affects their mental health as well as their physical development. Furthermore, positive economic conditions pave the way for an improved environment, better nutrition, higher-quality education, elevated social status, more friends, reduced feelings of loneliness, and increased social support and trust and all of these positively contribute to psychological well-being. 

Therefore, based on the conviction that early interventions can be protective and screening is needed to determine the proper intervention, this study aims to investigate the relationship between psychological well-being, loneliness, social support and social trust, all of which affect the psychological health of children living in economically disadvantaged families. 

To this end, answers to the following questions were sought.

1. Is there a significant relationship between the loneliness, social support, social trust and psychological well-being of the children from low-income families?
2. Do the feelings of loneliness, social support and social trust of the children from low-income families significantly predict their psychological well-being?

## 2. Method

### 2.1. Participants 

The present longitudinal study aimed to investigate the development and outcomes of low-income children over three waves of data collection. The initial sample consisted of 256 low-income children attending an elementary school in Türkiye, which is located in an economically disadvantaged neighborhood. Informed consent was obtained from the parents or legal guardians of all participating children, and ethical guidelines were strictly adhered to throughout the study. In the first wave of data collection (i.e., loneliness; in November 2022), 256 low-income children participated. During the second wave of data collection (i.e., social support and social trust; in December 2022), 58 children were unable to participate. The non-participation of these children was attributed to their absence from school. As a result, 198 low-income children remained in the study for the second wave. In the third and final wave of data collection (i.e., subjective well-being; in February 2023), 160 children participated. Among these, 38 children had not taken part in the first and second waves of the study. Also, six students were excluded because of poorly completed surveys. After accounting for the children who did not participate in either the second or third waves, the longitudinal study’s final sample consisted of 154 (49% female) low-income children. These 154 children demonstrated consistent participation across all three waves of data collection. The children were in the age range of 11 to 14 years (*M* = 12.75, *SD* = 0.73). The study was approved by the institutional review board. In addition to the location of the school, household incomes were obtained through a question. The participants in this study reported household incomes lower than 10,000 liras (11.7% = lower than 5500 ₤; 49.4% = 5001-to-8000 ₤; 39% = 8001-to-10,000 ₤). In June, the hunger threshold, which signifies the monetary amount necessary to prevent a four-person family from experiencing starvation for an entire month, was 10,360 liras (www.turkis.org.tr, accessed on 15 June 2023).

### 2.2. Measures

Loneliness. The Loneliness Scale is a three-item self-report measure (e.g., “I feel lonely”) utilized to assess feelings of loneliness [41]. The items were scored using a 5-point scale ranging from strongly disagree (1) to strongly agree (5). Previous research indicated that the scale had an adequate internal reliability estimate with Turkish children and adults [42]. In the first wave, the scale was administered to participants. 

Social Trust. Social trust was measured using the Trust Scale, a three-item self-report scale (e.g., “I can trust people in my society”) [41,43]. All items were responded to using a 5-point scale ranging from strongly disagree (1) to strongly agree (5). The internal reliability estimate of the scale for Turkish children was found to be adequate [42]. In the second wave, the scale was administered to participants.

Social support. The Social Support Scale is a three-item self-report instrument (e.g., “There are people who give me support and encouragement”) utilized to assess participants’ social support [41]. The scale items were scored based on a 5-point scale ranging from strongly disagree (1) to strongly agree (5). Previous research has shown that the scale had an adequate internal reliability estimate with Turkish children [42]. In the second wave, the scale was administered to participants.

Subjective well-being. Subjective well-being was measured utilizing the Subjective Well-being Scale, a nine-item self-report measure (e.g., “In most ways my life is close to my ideal”, “I feel positive most of the time”) designed to assess the cognitive (i.e., life satisfaction) and emotional components (i.e., positive and negative feelings) of subjective well-being [41,43]. The internal reliability estimates were between adequate and strong for Turkish children [42]. In the third wave, the scale was administered to participants. After reversing negative feeling items, subjective well-being was computed. 

### 2.3. Data Analyses

The initial phase of the study involved examining the descriptive statistics (e.g., mean, SD) and assessing the assumptions for the analyses. To assess the normality assumption, kurtosis and skewness scores were considered [44,45], while correlation estimates were used to explore the relationships between the measures. Subsequently, a mediation model was employed to investigate the potential mediating role of social support (wave 2) and social trust (wave 2) in the link between loneliness (wave 1) and children’s subjective well-being (wave 3). The PROCESS macro v4.2 (Model 4) in SPSS [46] was utilized for conducting this analysis. To determine the significance of indirect effects, the researchers employed the bootstrap method with 95% confidence intervals and 5000 resamples [46,47]. All statistical analyses were conducted using SPSS v27.

## 3. Results

Descriptive statistics and correlation results for the study variables are shown in Table 1. The analysis of skewness and kurtosis values indicated that all the measures utilized in the study exhibited relatively normal distributions [46,47]. Skewness and kurtosis scores were between −0.79 and 0.36, as seen in Table 1. Additionally, the study showed adequate-to-strong internal reliability estimates for the measures in the study, ranging from 0.74 to 0.91.

As shown in Table 1, the correlation results revealed that loneliness exhibited negative associations with social trust, social support and subjective well-being. On the other hand, social trust showed positive associations with and subjective well-being. Similarly, social support demonstrated a positive association with and subjective well-being among low-income children.

After conducting descriptive statistics and correlation analyses for the study variables, the proposed mediation model was employed to investigate the mediating role of social support (wave 2) and social trust (wave 2) in the link between loneliness (wave 1) and children’s subjective well-being (wave 3) utilizing the PROCESS macro v4.2 (Model 4) in SPSS. The unstandardized coefficients for the mediation model are presented in Table 2.

**Figure 1:**
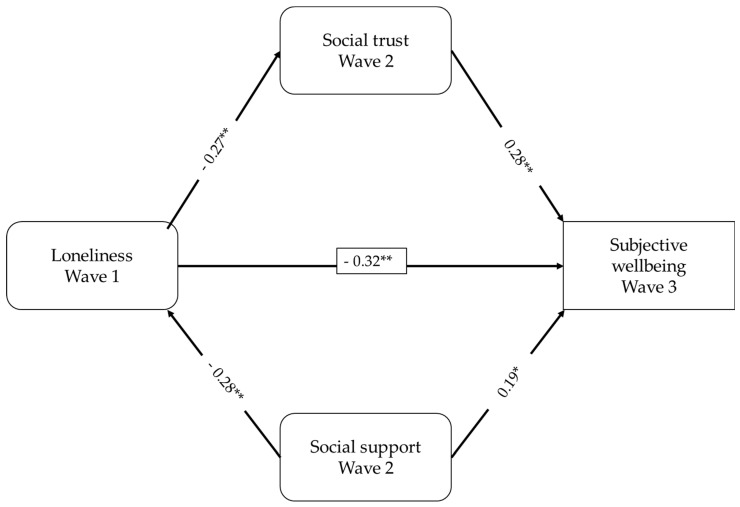
The mediation model indicating the longitudinal associations between the variables. Note. * *p* < 0.05, ** *p* < 0.001.

The results from the mediation model revealed significant and negative predictive effects of loneliness on social trust, social support, and subjective well-being among low-income children. Moreover, subjective well-being was predicted by both social support and social trust. Social support and social trust mediated the link between loneliness and subjective well-being. Loneliness was found to account for 7% of the variance in social trust and 8% of the variance in social support. When considered together, loneliness, social support, and social trust jointly explained 36% of the variance in subjective well-being. These findings indicate that loneliness not only directly predicted subjective well-being but also had an indirect effect through social support and trust as mediators in the relationship between loneliness and subjective well-being in the context of low-income children. The standardized effects of the mediation model are shown in Figure 1.

## 4. Discussion

The main purpose of the current study is to determine whether there is a significant relationship between loneliness, social support, social trust and psychological well-being of the children from low-income families. The interaction of these factors and their effects on children’s psychological health can determine the overall quality of their life. The feelings of loneliness, social support and social trust of the children from low-income families can significantly affect their psychological well-being. Loneliness can result from social isolation and a lack of social support [48]. Children from low-income families may experience feelings of loneliness due to their limited participation in social activities and limited social connections [49]. These variables are affected by individual factors, social structure, social interactions and cultural values [37]. The results obtained in the current study prove that there is a significant negative correlation between loneliness and psychological well-being. In other words, as the loneliness of the child increases, his/her psychological well-being deteriorates. Social support ensures that children have reliable and supportive relationships [50]. For children from low-income families, having a strong social support network can help them cope with adverse conditions and contribute positively to their psychological well-being. Social support can support psychological well-being by promoting positive emotional experiences. Recent studies have demonstrated the impact of social support in reducing loneliness [51,52]. However, the current study also assumes that social trust decreases loneliness and positively contributes to children’s well-being. Social trust refers to an individual’s trust and belief in other people and society. For children of low-income families, a lack of social trust can lead to difficulties in establishing relationships with others [53]. This can negatively affect children’s psychological well-being and increase their loneliness.

## 5. Conclusions

In conclusion, this study revealed that social trust and social support can reduce loneliness and thus improve children’s psychological well-being, and this result is consistent with the existing literature [54,55]. 

Furthermore, studies in the literature have revealed that loneliness is an undesirable condition and there are two main approaches to overcoming loneliness: rebuilding existing social networks and forming new social connections by making new friends [51]. This is indeed evidence showing that when children are socially supported and trust the people around them, they will feel better about themselves. According to Pettigrew [56], social support can help overcome the problems caused by loneliness. The current study also shows that social trust and social support are important needs for such children. Poverty and low levels of education were found to be associated with the effects of social support in the age groups studied [57]. For example, the need for competence and autonomy of a child who is psychologically well and happy is often met because of his/her high position in the social hierarchy. This makes the child feel more confident in himself/herself and his/her environment. Moreover, when sufficient social support is available, the likelihood of the child feeling lonely is reduced as he/she is content and happy. Thus, his/her need for relationships is fulfilled. Thus, it can be proposed that by enhancing the feelings of social support and social trust among children from low-income families, their levels of loneliness can be reduced, and their psychological well-being can be significantly improved. In this context, social services to be provided with educational and community support are of great importance in terms of meeting the social and emotional needs of children.

## 6. Suggestions

In this context, it is of great importance to provide appropriate solutions to children’s social and emotional needs through social services, education and community support.

In line with the general conclusion reached based on the research findings, the following can be suggested.

Recommendations for researchers based on the research findings:

- A comparative study can be planned in terms of loneliness, feelings of social support and social trust, and psychological well-being of children from low-income families and children from high-income families.
- More variables that may affect the psychological well-being of children from low-income families can be examined.
- The research can be conducted again on children living in different regions.
- New studies comparing the loneliness of female and male children between regions can be planned by keeping the sample size higher.
- Conducting comprehensive profile studies with multi-stakeholder research groups that reveal the quantitative and qualitative dimensions of the relationship between early childhood education and care and psychological well-being in low-income families.
- Childhood is a critical period in terms of personality development and spiritual development. In this period, children are negatively affected by conflicts in the family (Öngider, 2013). Therefore, in-depth research on the relationship between marital harmony/couple harmony and children’s psychological well-being levels can be planned.
- With the changing age, information technologies have also entered a new stage and digitalization has accelerated in the education platform as in every platform. In this rapidly digitalizing new world order, relational and experimental studies can be planned on the psychological well-being levels of children with poor economic status, their parents and teachers, and studies with suggestions such as developing practices for the development and protection of psychological health.

Suggestions for practitioners

Suggestions for practitioners to increase children’s psychological well-being, social support and social trust and to decrease the feeling of loneliness:

- The results of this study showed that social support, social trust, loneliness and psychological well-being are related. Improving the relationships of children from low-income families with their friends, peers and adult generations (mother, father, teachers, relatives) will provide both individual and social benefits. Based on this, creating opportunities and conditions that will increase children’s social support and trust, reduce their feelings of loneliness and increase their psychological well-being can be included in education and government policies.
- As the quality of life decreases, psychological well-being also decreases (Arslan, 2023). Therefore, relevant institutions should increase their efforts to take physical and psychological measures to improve the quality of life of children and families.
- In order to understand children’s emotions and positively support their psychological well-being, training can be prepared for mothers and fathers, who are the most important individuals in children’s development. These trainings can start with low-income families.
- Creating effective platforms and channels for low-income families to obtain information on the acquisition and protection of psychological well-being in early childhood.
- The materials prepared in the process of informing families can be made available to more people on social media information-sharing channels and platforms.

## Figures and Tables

**Table 1:** Descriptive statistics.

	*Mean*	*SD*	Skew.	Kurt.	α	1	2	3	4
1. Loneliness W1	7.34	3.21	0.36	−0.64	0.75	-	−0.27 **	−0.28 **	−0.46 **
2. Social trust W2	8.52	2.61	−0.25	−0.27	0.81		-	0.53 **	0.47 **
3. Social support W2	11.51	2.83	−0.79	0.15	0.74			-	0.43 **
4. Subjective well-being W3	11.09	7.04	0.11	−0.01	0.89				-

** *p* < 0.001.

**Table 2:** Unstandardized coefficients for the mediation model.

	Consequent			
	*M*_1_ (Social Trust)			
Antecedent	Coeff.	*SE*	*t*	*p*
*X* (Loneliness)	–0.122	0.06	–3.48	<0.001
Constant	10.14	0.51	19.93	<0.001
	*R*^2^ = 0.07 *F* = 12.10; *p* < 0.001			
	*M*_2_ (Social support)			
Antecedent	Coeff.	*SE*	*t*	*p*
*X* (Loneliness)	–0.24	0.07	–3.53	<0.001
Constant	13.29	0.55	24.12	<0.001
	*R*^2^ = 0.08 *F* = 12.46; *p* < 0.001			
	*Y*_1_ (Subjective well-being)			
Antecedent	Coeff.	*SE*	*t*	*p*
*X* (Loneliness)	–0.72	0.15	–4.77	<0.001
*M*_1_ (Social trust)	0.75	0.20	3.62	<0.001
*M*_2_ (Social support)	0.48	0.19	2.46	0.014
Constant	5.20	2.65	1.96	0.051
	*R*^2^ = 0.36 *F* = 28.79; *p* < 0.001			
Standardized indirect effects				
Paths	Effect	*SE*	BootLLCI	BootULCI
Total indirect effect	–0.13	0.04	–0.22	–0.05
Loneliness → Social trust → Well-being	–0.08	0.03	–0.15	–0.02
Loneliness → Social support → Well-being	–0.05	0.02	–0.11	–0.01

Note. Number of bootstrap samples: 5000. *SE* = standard error. Coeff = unstandardized coefficient. Subjective well-being = life satisfaction + positive feelings − negative feelings.
